# Paracrine ADP Ribosyl Cyclase-Mediated Regulation of Biological Processes

**DOI:** 10.3390/cells11172637

**Published:** 2022-08-24

**Authors:** Cecilia Astigiano, Andrea Benzi, Maria Elena Laugieri, Francesco Piacente, Laura Sturla, Lucrezia Guida, Santina Bruzzone, Antonio De Flora

**Affiliations:** Department of Experimental Medicine (DIMES), Section of Biochemistry, University of Genova, 16132 Genova, Italy

**Keywords:** CD38, connexin 43, NAD^+^, cyclic ADP-ribose

## Abstract

ADP-ribosyl cyclases (ADPRCs) catalyze the synthesis of the Ca^2+^-active second messengers Cyclic ADP-ribose (cADPR) and ADP-ribose (ADPR) from NAD^+^ as well as nicotinic acid adenine dinucleotide phosphate (NAADP^+^) from NADP^+^. The best characterized ADPRC in mammals is CD38, a single-pass transmembrane protein with two opposite membrane orientations. The first identified form, type II CD38, is a glycosylated ectoenzyme, while type III CD38 has its active site in the cytosol. The ectoenzymatic nature of type II CD38 raised long ago the question of a topological paradox concerning the access of the intracellular NAD^+^ substrate to the extracellular active site and of extracellular cADPR product to its intracellular receptors, ryanodine (RyR) channels. Two different transporters, equilibrative connexin 43 (Cx43) hemichannels for NAD^+^ and concentrative nucleoside transporters (CNTs) for cADPR, proved to mediate cell-autonomous trafficking of both nucleotides. Here, we discussed how type II CD38, Cx43 and CNTs also play a role in mediating several paracrine processes where an ADPRC^+^ cell supplies a neighboring CNT-and RyR-expressing cell with cADPR. Recently, type II CD38 was shown to start an ectoenzymatic sequence of reactions from NAD^+^/ADPR to the strong immunosuppressant adenosine; this paracrine effect represents a major mechanism of acquired resistance of several tumors to immune checkpoint therapy.

## 1. Introduction

ADP-ribosyl cyclases (ADPRCs) are a family of evolutionarily conserved signaling enzymes with unique properties [1,2,3,4,5,6,7,8,9,10,11,12,13,14,15]. They show low substrate specificity (mostly NAD^+^ and NADP^+^) and eight naturally occurring enzyme products, all Ca^2+^-active, including Cyclic ADP-ribose (cADPR), ADP-ribose (ADPR), and controversially, nicotinic acid adenine dinucleotide phosphate (NAADP^+^) [16,17,18,19,20,21,22]. In mammals, the best characterized ADPRC, CD38, is a single-pass transmembrane protein that exists in two opposite orientations, the dominant type II glycosylated protein (an ectoenzyme) and a type III non-glycosylated protein, mainly functioning in the cytosol [23,24]. Another ADPRC ectoenzyme and CD38 homolog is the glycosylphosphatidylinositol-anchored protein BST1/CD157 in bone marrow (BM) stromal cells [25,26]. SARM1, a new important member of the ADPRC family, is an intracellular enzyme largely expressed in neurons, which although being structurally unrelated, shares catalytic activities with CD38 [27,28,29].

The early detection of type II CD38 led to identify a topological paradox, inherent to the apparent unavailability of substrate NAD^+^ to the ectocellular catalytic site and to the fact that the product cADPR should not be available to its intracellular endoplasmic reticulum (ER) ryanodine (RyR) receptors/stores that elicit Ca^2+^ mobilization [30,31,32,33,34,35,36]. The same topological inconsistencies concerning the plasma membrane appeared to also challenge the functioning of the fraction of type II CD38 present in cellular organelles as well as in intracellular vesicles, both endocytotic and exocytotic. The topological paradox of type II CD38/NAD^+^/cADPR system was addressed by two approaches: on the one hand, connexin 43 (Cx43) hemichannels were found to mediate an equilibrative (i.e., transport direction follows the concentration gradient) Ca^2+^-regulated transport of intracellular NAD^+^ to the ectocellular active site of CD38 [37,38]. On the other hand, different systems of concentrative (i.e., transport direction is against the gradient) nucleoside transporters (CNTs) proved to mediate inverse access of extracellular cADPR to the RyRs [39]. Therefore, a functional crosstalk between NAD^+^ and cADPR transporters was characterized that allows for the variable traffic of signal metabolites with the regulation of [Ca^2+^]_i_ levels [32,34,39,40,41].

Recent studies by H.C. Lee and Y.J. Zhao, who identified and characterized type III CD38 as a major cADPR synthesizer in the cytosol [23,24,42], and by U.H. Kim, who demonstrated the coordinated involvement in the synthesis of cADPR and NAADP^+^ of both type II and type III CD38 in different organelles [43], substantially clarified cell-autonomous mechanisms of ADPRC-mediated Ca^2+^ regulation.

**Figure 1:**
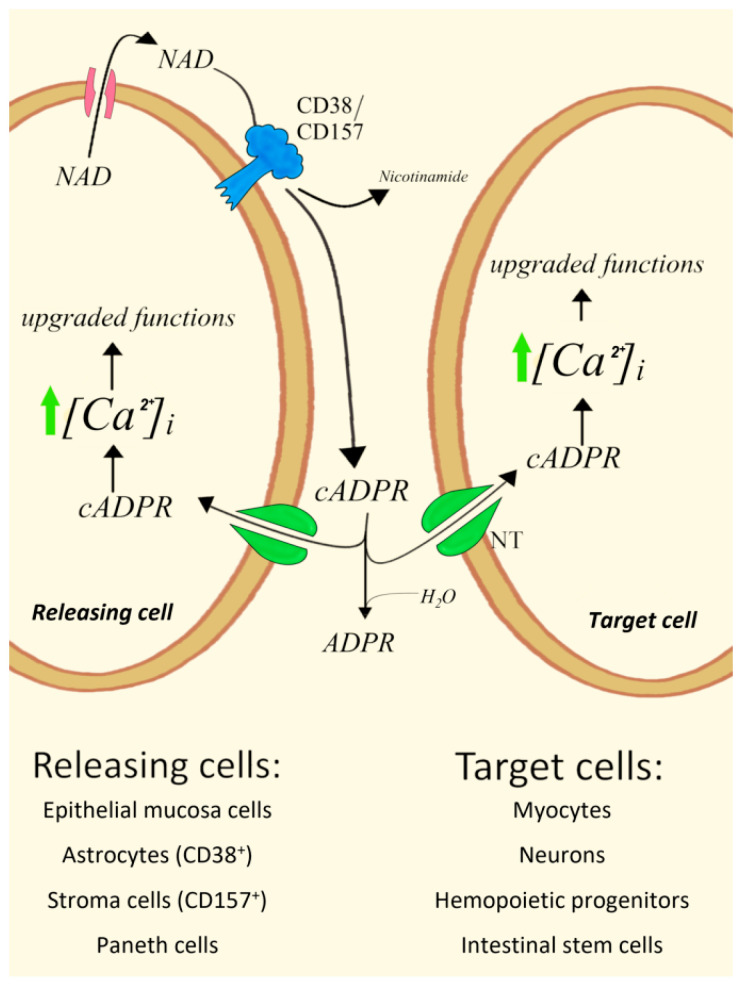
General scheme of cADPR-mediated paracrine interactions between a cADPR-generating and a cADPR-responsive cell. NT, nucleoside transporters.

Here, we present evidence that type II CD38 also plays a major role in paracrine processes mostly driven by cADPR as an intercellular signal metabolite through the same transporters (Cx43 and NTs) responsible for autocrine processes. Specifically, several cell systems were described and reconstructed, where a CD38^+^ (or CD157^+^) and Cx43^+^ cell provides cADPR to a different cell, even ADPRC^low^ or ADPRC^−^, expressing the promiscuous NTs involved in concentrative cADPR internalization and cADPR-responsive RyRs (Figure 1). These cell systems include: (a) epithelial mucosa cells/smooth myocytes in bovine trachea, (b) homotypic CD38^+^/CD38^−^ co-cultures of 3T3 murine fibroblasts, (c) rat astrocytes/hippocampal neurons co-cultures, (d) CD38^+^ or BST1^+^ stroma cells/human hemopoietic progenitors (HP) in bone marrow (BM), both in vitro and in vivo, and (e) CD157^+^ Paneth cells/intestinal stem cells.

In addition, a related breakthrough in the CD38 field was achieved by F. Malavasi’s group, who showed that type II CD38 can start a totally ectoenzymatic chain of reactions eventually leading to extracellular adenosine generation [44,45,46,47,48,49,50]. Based on these data, D.L. Gibbons recently demonstrated that in the microenvironment of several solid tumors (TME), the upregulation of CD38 is responsible for adenosine-mediated immunosuppression via adenosine receptor signaling on cytotoxic T lymphocytes [51]. This CD38-mediated paracrine effect is a major mechanism of acquired resistance of tumors treated with immune checkpoint inhibitors (PD-1, Programmed cell death protein 1/PD-L1 blocking antibodies) [51,52,53].

Therefore, due to its ectoenzymatic nature, type II CD38 seems to play major roles in a vast number of pathophysiological conditions amenable to type II CD38-targeted therapies.

## 2. Type II CD38 and NAD^+^ and cADPR Transporters

The topological paradox that characterizes the dynamic traffic of NAD^+^ and cADPR in type II CD38-expressing cells does not exist for non-ectoenzymatic ADPRCs, such as type III CD38 and SARM1. Accordingly, as widely reported by H.C. Lee and Y.J. Zhao in recent years [23], the regulation of intracellular cADPR-synthesizing activity occurs largely in type III CD38 and in the variable expression and control of mechanisms that modulate the access of NAD^+^ substrate to type II CD38 and the translocation of cADPR product to RyRs receptors/stores [23,24,32,34]. Once more, it should be emphasized that these mechanisms can operate at the plasma membrane level but also in intracellular membrane vesicles (either exocytotic or endocytotic) and in cell organelles. Concerning the vesicles, we reported years ago that the exposure of CD38^+^ Namalwa cells to NAD^+^ or to GSH induces an extensive internalization of plasma membrane type II CD38, with the import of NAD^+^/cADPR metabolism from the plasma membrane to endocytotic membrane vesicles [54]. Moreover, a novel negative feedback mechanism was discovered in Namalwa cells, where the prolonged activation of RyRs results in lysosomal degradation of the cADPR-producing ectocellular CD38 [55]. Finally, we demonstrated that de novo ectocellular (i.e., type II) CD38 expression in CD38^−^ 3T3 and HeLa cells, arguably through exocytotic membrane vesicles, entails intracellular NAD^+^/NADH consumption and cADPR accumulation. These metabolic changes were accompanied by the depletion of thapsigargin-sensitive calcium stores, an increase in cytosolic [Ca^2+^]_i_ levels and a higher proliferation rate [56].

The search for the nucleotide transporters responsible for NAD^+^ and cADPR traffic was mostly limited to the plasma membrane. Here, we first identified Cx43 hemichannels as an equilibrative NAD^+^ translocation system and hypothesized that this protein may be causally involved in triggering NAD^+^-related autocrine as well as paracrine processes [37]. Remarkably, the conversion of NAD^+^-permeable Cx43 to the phosphorylated NAD^+^-impermeable form occurs via Ca^2+^-stimulated protein kinase C (PKC). Therefore, a self-regulatory loop emerged in CD38^+^ 3T3 cells whereby high [Ca^2+^]_i_ restricts further Ca^2+^ mobilization by cADPR via the protein kinase C (PKC)-mediated disruption of the functional Cx43-CD38 crosstalk [38].

Interestingly, the field of Cx43 phosphorylation has revealed a high number of different kinases being involved at several sites, accounting for as many as 21 phosphorylation sites [57], in different cell types and with a number of structural consequences on connexin functions [58,59]. However, the property of NAD^+^ permeability received little attention, with the exception of a study by U.H. Kim’s group. These authors reported that the physiological stimulation of J774A.1 murine macrophage cell line activates protein kinase A (PKA), which results in Cx43 phosphorylation, the enhanced leakage of NAD^+^ from the hemichannels, CD38-mediated extracellular conversion of NAD^+^ to cADPR, and the import of cADPR across Cx43 and increased Ca^2+^ via RyRs [60]. These findings open the possibility of an opposite correlation between PKA and PKC in upregulating and downregulating NAD^+^ translocation, respectively, across CX43, and perhaps in driving opposite NAD^+^ fluxes in some cell types. The redundancy of kinases and phosphatases acting in the Cx43 protein [57,58] might explain these different findings on NAD^+^ efflux/influx in the plasma membrane as well as in intracellular vesicles and organelles. In any case, the complexity in the field requires further investigations to address the mechanisms of Cx43-mediated bidirectional fluxes of NAD^+^ and of cADPR import into the cells.

**Figure 2:**
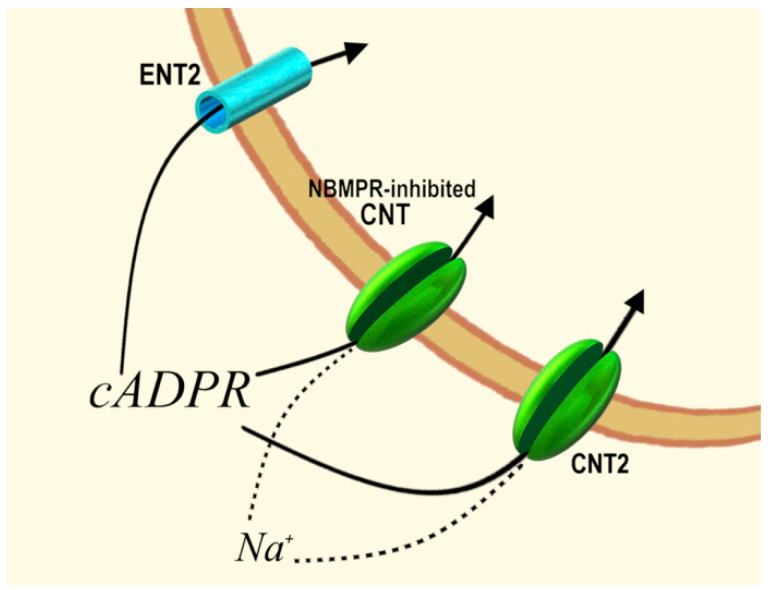
Uptake of extracellular cADPR through equilibrative and concentrative cADPR/nucleoside transporters. ENT2, equilibrative nucleoside transporter 2; CNT2, concentrative nucleoside transporter 2; NBMPR-inhibited CNT, nitrobenzoylthioinosine-inhibited concentrative nucleoside transporter.

We also investigated the identity and the properties of cADPR transporters potentially involved in the internalization of extracellular cADPR in the same (autocrine) or in adjacent (paracrine) cells. Several complementary approaches, i.e., use of selective inhibitors or experimental conditions (abrogation of Na^+^-dependent active symport processes, analysis of RNA transcripts from different cell types, transient transfection experiments), demonstrated that cADPR influx can occur across redundant proteins responsible for either equilibrative or concentrative transmembrane translocation [32,34]. These were identified with promiscuous nucleoside transporters, including equilibrative (ENT2) and concentrative (CNT2, CNT3, a nitrobenzoylthioinosine (NBMPR)- and dipyridamole (DP)- inhibitable NT) [39,40,41]. Other known ENTs and CNTs were unable to translocate cADPR. The transport activity proved to be inhibitable by uridine, guanosine and inosine. Moreover, the cADPR transport by the CNTs was found to be a Na^+^/cADPR symport mechanism. Clearly, the potent cADPR-transporting activity of the CNTs suggests a major role of these proteins in allowing cADPR transport against largely unfavorable concentration gradients, a in function that is unlikely ENTs (Figure 2).

## 3. Type III CD38

Approximately 20 years after his seminal discovery of CD38 as a multifunctional enzyme converting NAD^+^ to cADPR and ADPR and NADP^+^ to NAADP^+^ [2,3,4,11], Hon Cheung Lee first hypothesized, and then clearly demonstrated, the occurrence of type III CD38 in several cell types. Together with Yong Juan Zhao, he reported that type III CD38 has a membrane orientation opposite to that of the ectoenzyme type II [14,15]. This groundbreaking result was in agreement with the hypothesis that CD38 should obey to the positive inside rule, stating that the more positively charged side of the two regions of a transmembrane protein is the side located in the cytosol [61]. This is the case of CD38, which explains a preferential expression of its type II form, as found in most cells, but leaves the possibility of a lower expression as the type III. Sophisticated experiments of site-directed mutagenesis showed that a basal expression of native type II can revert to a large proportion of fully catalytically active type III CD38 [14,15]. Another way to activate native type III CD38 was reported by U.H. Kim in mouse lymphokine-activated killers (LAK) cells [62]. Here, interleukin 8 (IL-8) triggered an oxidative mechanism of H_2_O_2_-induced-activation of type III CD38 with an enhanced generation of cADPR.

As anticipated, the fact that type III CD38 has its catalytic site facing the cytosol, and is therefore fully available to its substrates NAD^+^ and NADP^+^, rules out the possibility of topological challenges to its intracellular functioning. Instead, the regulation of its activity seems to be amenable to canonical mechanisms through allosteric modulators, protein-protein modifications, or CD38 protein expression levels. One example is the cytosolic Ca^2+^-binding protein CIB1, which was shown to bind to the CD38 active site through its N terminus both in vitro and in live cells. Interestingly, there was a direct correlation between cellular cADPR and CIB1 levels [42].

Another mechanism that seems relevant to the regulation of type III CD38 is the activity of cellular chaperones. H.C. Lee and Y.J. Zhao reported that Hip and Hsp90 are involved in the correct folding of type III CD38, while Hsc70, DNAJA1 and DNAJA2s are able to target this protein to lysosomal degradation [63]. Admittedly, this is a complex mechanism, depending on the nature of the client proteins and on the constellation of the various co-chaperones, but it seems to confirm the physiological relevance of type III CD38 regulation through quantitative variations in its cellular expression.

Recent findings by H.C. Lee and Y.J. Zhao [23], as well as by U.H. Kim, document the different roles of both type II and type III CD38 and their effects on Ca^2+^ homeostasis through compartmentalized cADPR and NAADP^+^ formation. The Lee/Zhao team critically analyzed the role of type II CD38 in cellular cADPR generation and obtained different lines of evidence against this possibility, while implicating type III CD38/C1B1 complex as responsible for most cADPR-synthesizing activity [42]. Specifically, using a technique exploiting an anti-CD38 nanobody-based photoaffinity labeling, they discovered that cell surface CD38 interacts with the transferrin receptor CD71 and that the CD38-CD71 complex is present naturally in the myeloma cell line LP-1 [42]. They also addressed the subcellular site and the features of the CD38 activity responsible for NAADP^+^ formation. The underlying approach was the use of anti-CD38 nanobodies which triggered surface CD38 internalization to endolysosomes and nicotinic acid-dependent NAADP^+^ synthesis [64].

As mentioned, in an earlier investigation, we found that extracellular NAD^+^ induced a ligand-mediated internalization in human Namalwa B cells in non-clathrin-coated vesicles [54]. The stimulus for CD38 endocytosis was therefore different from that used by H.C. Lee and Y.J. Zhao, who exploited anti-CD38 nanobodies to induce a clathrin-dependent endocytotic pathway [64]. Indeed, in our study the non-clathrin-mediated NAD^+^-dependent endocytosis of type II CD38 was found to downregulate the cADPR-synthesizing activity of plasma membrane CD38, which was largely imported in the cytosol [54].

Kim’s team investigated the LAK cells as a suitable system for studies on receptor-mediated Ca^2+^ signaling [43]. In fact, these cells express both type II and type III CD38 and are responsive to IL-8 stimulation, which ultimately result in cell migration through a complex transduction pathway involving three major Ca^2+^ signaling messengers, inositol 1,4,5-trisphosphate (IP3), cADPR and NAADP^+^ [43]. The first event in IL-8-stimulated LAK cells is the activation of phospholipase C beta2 (PLC-beta2) with a consequent biosynthesis of IP3 and Ca^2+^ release from ER via IP3 receptor (IP3R). This triggers a guanylate cyclase/cGMP/PKG cascade, resulting in the phosphorylation of MHCHA and p22phox, which induces internalization in early endosomes of type III CD38, whose cytosol-facing catalytic site leads to an increase in cADPR generation [43,65]. As previously mentioned, IL-8 also induces the NADPH oxidase 4 (Nox4)-mediated production of H_2_O_2_ in LAK cells, with consequent activation of type III CD38 through the formation of a Cys 164-Cys 177 disulfide bond and increased synthesis of cADPR [43]. The cADPR-dependent Ca^2+^ release from the ER via the RYRs stimulates adenylate cyclase, with formation of cAMP and activation of PKA and EPAC. IL-8 induces the activation of Rap1 through PKA and a complex is formed between PKA, EPAC and activated Rap1 with type II CD38 in the intraluminal environment of acidic lysosomes, representing the suitable site for CD38-catalyzed base exchange between nicotinic acid of NAAD and NADP^+^. The availability of NADP^+^ for NAADP^+^ generation is ensured by the transport of NADP^+^ from the cytosol to the endolysosomal space across Cx43 hemichannels [43].

To summarize, the mechanisms of IL-8 stimulation resulting in an enhanced migration of LAK cells involve a highly integrated transduction system where the Ca^2+^-related second messengers cooperate through a crosstalk occurring at different sites and at different times in these cells [43]. Readers are invited to view the complete schemes depicting the molecular mechanisms presented by the Kim’s lab [43]. The specific and unique hallmarks of this system are:

(a) CD38 catalyzes the intracellular synthesis of both cADPR and NAADP^+^.
(b) A cGMP-PKG cascade triggered by the [Ca^2+^]_i_ release from the ER through IP3 induces the internalization of type III CD38 from the plasma membrane into early endosomes with the generation of cADPR at their outer surface and the release of cADPR in the cytosol.
(c) cADPR and ER-mobilized Ca^2+^ activate a PKA-EPAC-Rap1 cascade driven by cAMP production that stimulates NAADP^+^ formation by type II CD38 inside acidic lysosomes.
(d) The type II-mediated generation of NAADP^+^ inside acidic organelles takes place through a nicotinic acid and nicotinamide base exchange between NAAD (an intermediate of NAD^+^ biosynthesis) and NADP^+^ (which is taken up from the cytosol across activated Cx43 hemichannels) [43].
(e) The time sequence of second messengers is IP3-cADPR-NAADP^+^ and the consequent [Ca^2+^]_i_ increases downstream of their binding to specific receptors (IP3, RyR and TPC, respectively) occur in a transient fashion (cADPR) followed by a long-lasting signal (NAADP^+^).

Thus, the ultimate players of this complex signaling system are the two forms of CD38 (type III and type II), localized in different but interacting organelles, as well as the transporters of the various nucleotides (NAD^+^, NADP^+^, cADPR), substrates and products of CD38 that circumvent topological hindrances to type II CD38 functioning in the cell. The resulting picture is a multilayered regulation of calcium homeostasis through the variable traffic of these signal metabolites that impact LAK cells migration [43].

While the discovery of type III CD38 significantly elucidated the mechanisms of the topological paradox, regulation in type II CD38, either at the plasma membrane or inside cellular organelles, occurs on the access of substrate NAD^+^ to its active site and of the product cADPR to RyRs [24]. The detailed mechanisms of the crosstalk between Cx43, the cADPR/nucleoside transporters and type II CD38 require further investigations.

Another topic that warrants adequate studies concerns the possible roles of the intracellular enzyme protein SARM1, in view of its catalytic activity responsible for cADPR and NAADP^+^ formation despite a completely different structure from CD38 [27,28]. Available pieces of evidence seem to indicate that SARM1 is a regulated cytosolic enzyme that impacts the homeostasis of cellular calcium by virtue of its cADPR-synthesizing activity, that quantitatively exceeds the one featured by CD38. It is quite interesting that CZ48 (a sulfo-araF-NMN), a cell-permeant synthetic inhibitor of CD38 and mimetic of NMN, is conversely an activator of SARM1 in several cell types [27]. Accordingly, the genetic ablation of NMN adenylyltransferase elevated cADPR formation through an increased availability of intracellular NMN. Therefore, the major pathway of NAD^+^ biosynthesis seems to be principally correlated to the NMN-dependent, SARM-1-mediated degradation of NAD^+^ and cADPR increase in cells.

## 4. Type II CD38 and CD157 cADPR-Synthesizing Ectoenzymes Mediate Paracrine Mechanisms

### 4.1. Epithelial Mucosa Cells and Smooth Myocytes in Bovine Trachea

**Figure 3:**
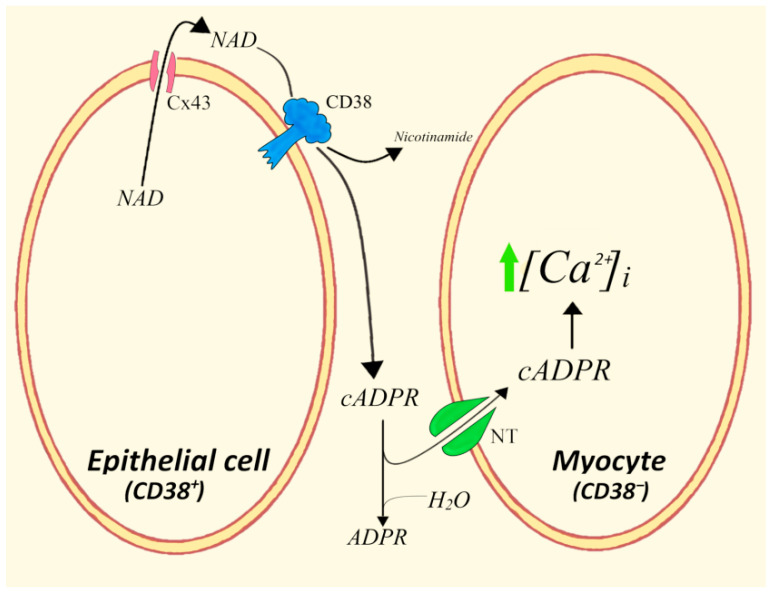
Paracrine roles of NAD^+^/cADPR from epithelial cells in eliciting Ca^2+^ responses in smooth myocytes. NT, nucleoside transporters; Cx43, connexin 43.

The first evidence for a paracrine exchange of cADPR between two different cell types was obtained before the identification and characterization of Cx43 as a NAD^+^ transporter and of CNTs as cADPR transporters. Thus, an intercellular crosstalk way demonstrated in bovine trachea where the NAD^+^ releasing and cADPR-generating CD38 in mucosal epithelium elicits an increase in [Ca^2+^]_i_ levels in cADPR-responsive smooth myocytes (Figure 3) both at baseline and after acetylcholine (Ach) stimulation [66]. Interestingly, the [Ca^2+^]_i_-related variations caused a significant increase in Ach-induced contraction in tracheal muscle strips, which was reduced by the cADPR antagonist 8-NH_2_-cADPR.

This unprecedented interplay between two different cell types was predicted to be a common mechanism of cADPR function in several mammalian tissues, which indeed proved to be the case. In this study, freshly isolated TSMs were incubated with intact mucosal strips. The mucosa-dependent increase in TSM [Ca^2+^]_i_ was prevented by the addition to the medium either of NAD^+^-glycohydrolase or of the cADPR-antagonist 8-NH_2_-cADPR, which indicated the involvement of both extracellular NAD^+^ and cADPR in the TSM calcium response to epithelial mucosa.

### 4.2. CD38^+^/CD38^−^ Cells in Homotypic Co-Cultures of 3T3 Fibroblasts

**Figure 4:**
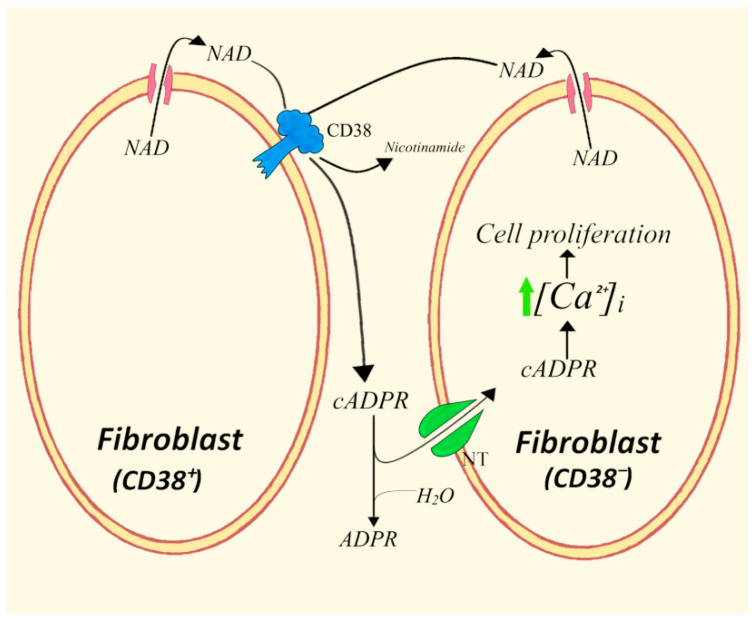
Uptake and effects of extracellularly generated cADPR by CD38^−^ 3T3 fibroblasts. NT, nucleoside transporter.

The discovery of Cx43 hemichannels as an equilibrative NAD^+^ transporter responsible for intracellular NAD^+^ efflux across the plasma membrane, as well as for cytosolic NAD^+^ transport to the intraluminal space of subcellular membrane vesicles, allowed the clarification of molecular mechanisms whereby NAD^+^ can reach the active site of the ectoenzyme CD38. An additional piece of information came from the demonstration that a topologically opposite process of cADPR translocation from either the outer cell surface or from the cytosol to the intraluminal environment of cell vesicles can take place through some concentrative, promiscuous transporters of nucleosides and cADPR [39,40,41]. A better focus on the paracrine mechanisms of traffic of NAD^+^ and cADPR was obtained by investigating different types of transwell co-cultures of CD38^+^ cells with CNTs- and RyR-expressing CD38^low^ or CD38^−^ cells (Figure 4). Using this simple system, it was possible to dissect the intra- and intercellular steps of the different roles CD38^+^ cells play in adjacent cells that sense and internalize cADPR [32,33,34]. A twofold considerable advantage of this approach was: (i) the addition of enzymes in the extracellular medium, which allowed NAD^+^ to be degraded as in the case of NAD^+^-glycohydrolase and converted NAD^+^ to cADPR by means of added ADPRc from Aplysia californica; (ii) the analysis of intracellular and extracellular metabolites (NAD^+^, cADPR, ADPR) in the two types of co-cultured cells treated with cADPR antagonists (8-Br-cADPR and 8-NH_2_-cADPR), or with Cx43 modulators (oleamide, anti-Cx43 deoxynucleotides, Cx43 sense deoxynucleotides) [39,40]. These analyses were integrated by assays of levels and kinetics of [Ca^2+^]_i_ and by evaluating the increases in cell proliferation of CD38^−^ fibroblasts co-cultured over CD38^+^ cells. Statistical analysis of values recorded with the two cell populations showed a shorter S phase of cell cycle for the CD38^−^ target population over CD38^+^ feeders as compared with the control over homologous CD38^−^ cells [67].

Therefore, a paracrine process emerged based on the Cx43-mediated release of NAD^+^, followed by its CD38-catalyzed conversion to ectocellular cADPR and eventual influx of cADPR into responsive adjacent cells to enhance their [Ca^2+^]_i_ levels and stimulate cell proliferation.

### 4.3. Bidirectional Astrocytes/Neurons Communications Involving the CD38/cADPR System and Glutamate

**Figure 5:**
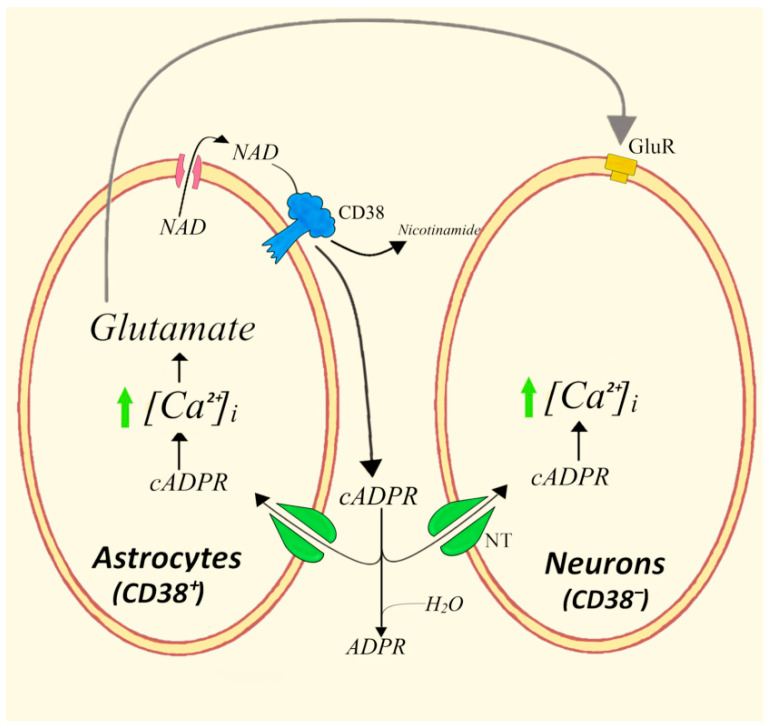
Paracrine/autocrine roles of NAD^+^/cADPR and glutamate in eliciting Ca^2+^ responses in astrocytes and neurons. GluR, Glutamate receptor; NT, nucleoside transporters.

For several years, a number of studies have documented the occurrence of bidirectional communications between astrocytes and neurons, strengthening the idea of an important role of astrocytes in the physiology of the nervous system [68]. Using both contact co-cultures of hippocampal neurons plated over hippocampal astrocytes and transwell co-cultures of these cell types without any physical contact between them, we provided evidence of complex molecular interactions centered on the CD38/NAD^+^/cADPR system and glutamate system [69]. Specifically, CD38^+^ and Cx43^+^ astrocytes proved to release NAD^+^ and to respond to either extracellular NAD^+^ or cADPR with intracellular calcium increases, resulting in the enhanced formation of glutamate according to a cell-autonomous mechanism (Figure 5). A first paracrine extension of calcium responses was observed by measuring the astrocyte-generated fraction of cADPR, which induced a weak [Ca^2+^]_i_ increase in adjacent neurons. The consequent neuronal Ca^2+^ response proved to be further strengthened by astrocyte-released glutamate through an additional paracrine and more efficient astrocyte–neuron crosstalk. Further experiments demonstrated that the co-culture of astrocytes with neurons triggers a feed-forward paracrine mechanism causally mediated by neurons through glutamate and resulting in a significant overexpression of astrocyte CD38, with the consequent generation of intracellular cADPR and increased Ca^2+^ levels [70].

Therefore, the CD38/NAD^+^/cADPR/Ca^2+^/glutamate system accounts for a network of bidirectional communications between astrocytes and neurons that involves both autocrine and paracrine mechanisms and the enhanced traffic of signal metabolites and neuromodulators connecting the two cell types. The role of cADPR as an important second messenger involved in Ca^2+^ signaling in neurons had been previously demonstrated by the finding, that in rodent NG108-15 neuroblastoma cells, the overexpression of human CD38 can contribute to acetylcholine-induced Ca^2+^ signaling [71]. Moreover, acetylcholine was reported to stimulate cADPR formation via M1 subtype muscarinic receptors in rat superior cervical ganglion [72].

A major contribution by H. Higashida’s team on the CD38/NAD^+^/cADPR system in the brain was the demonstration that adult CD38^−/−^ female mice show major defects in maternal nurturing and that CD38^−/−^ male mice have defects in social behavior [73]. These abnormalities were causally related to a deficiency of cADPR that resulted in the low release of oxytocin in plasma and cerebrospinal fluid, although these were found in high levels in the hypothalamus and neurohypophysis nerve endings [74]. Oxytocin is released in oxytocin-producing neurons in the hypothalamus via the cADPR-activated Ca^2+^ mobilization from RyRs that follows Ca^2+^ influx (CICR-calcium induced calcium release) [75]. The link between biochemical mechanisms in oxytocin-synthesizing and -releasing cells and behavioral properties is provided by the CD38/NAD^+^/cADPR/Ca^2+^ system, which is critical for social recognition [74]. This remarkable correlation is strongly indicated by the fact that in humans, single-nucleotide polymorphisms in CD38 may represent risk factors for autism spectrum disorders by downregulating oxytocin functions [73,75].

This research field received recent and important contributions obtained by investigating the different roles of the cADPR-synthesizing ectoenzymes CD38 and CD157 in social behavior phenotypes during aging in mice. Thus, using specific KO strains, a marked age-dependent anxiety-related behavior was observed in CD157 KO mice and only a slight change in CD38 KO mice [76]. Research on this topic is in progress, and specifically, the impact of either enzyme defect on cADPR/Ca^2+^/oxytocin needs to be clarified [77]. Additionally, the possible occurrence of paracrine cADPR-mediated interactions involving the two ectoenzymes CD38 and CD157 and oxytocin/oxytocin receptors is still unknown [78], although it is suggested by the distinct types of cells expressing either ecto-ADPRC and cells synthesizing oxytocin/oxytocin receptors.

The deep complexity of cADPR/Ca^2+^ interactions in the central and peripheral nervous system is also documented by the frequent occurrence of peripheral neuropathy in cancer patients treated with a number of chemotherapy drugs. Calcium dysregulation represents a critical event of axonal degeneration. A recent report indicates that cADPR generated by the intracellular ADPRC SARM1 increases intra-axonal Ca^2+^ and promotes axon degeneration caused by the drug paclitaxel [79,80]. Genetic depletion of SARM1, but not of CD38, in sensory neurons prevented both the axonal degeneration and elevation of cADPR induced by paclitaxel. Moreover, the permeant cADPR antagonist 8-Br-cADPR was able to block axonal degeneration to a complete extent, but without totally abrogating the underlying paclitaxel-induced increase in axonal calcium. Thus, apparent inconsistencies suggest that cADPR is only one of different mediators of paclitaxel-induced axon degeneration or that the site of cADPR production, i.e., the cytosol in the case of SARM1 activation or at the level of plasma membrane or intracellular membranes by the ectoenzyme CD38 may be mechanistically relevant to axonal degeneration.

### 4.4. ADPRC^+^ Stroma Cells/Human Hemopoietic Progenitors (HPs)

**Figure 6:**
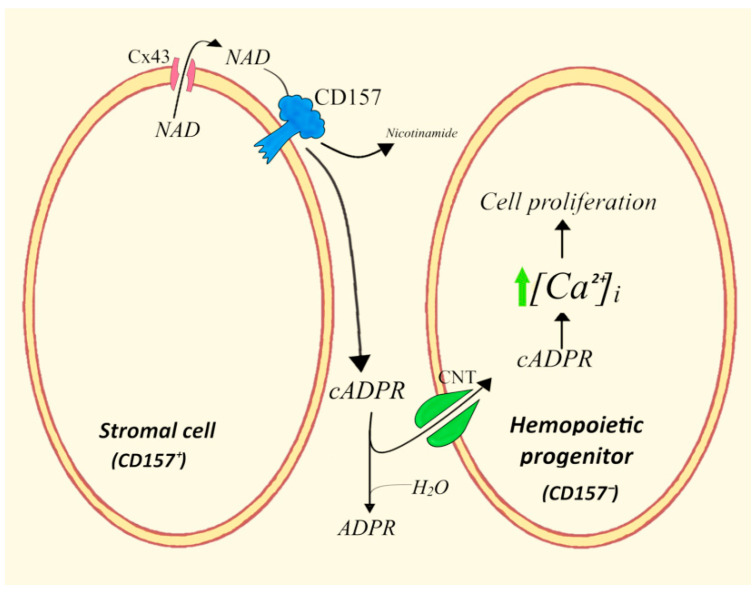
Paracrine interactions between CD157-expressing stromal cells and human hemopoietic cells. The stroma-produced cADPR determines a calcium-mediated expansion of human hemopoietic cells. CNT, concentrative nucleoside transporter.

The enhanced, cADPR/Ca^2+^-stimulated proliferation of 3T3 fibroblasts [67] led to an investigation of whether similar effects of the NAD^+^/cADPR axis could take place in other cell types. Indeed, the exposure of human-committed HPs, as colony-forming cells (CFC), to cADPR for 24 h caused a significant increase in colony output and colony size [81]. The priming effect of extracellular cADPR was an enhanced cell growth for multiple generations and over several weeks. Priming involves the influx of extracellular cADPR and the subsequent increase in [Ca^2+^]_i_ in the HP, leading to CFC expansion (Figure 6).

In order to identify the possible source of cADPR as a pro-hemopoietic cytokine in the bone marrow (BM) microenvironment, the HPs were cocultured for 5 weeks with stromal cell lines in different conditions using a transwell setting. Moreover, the most immature HPs, the long-term culture-initiating cells (LTC-IC), were studied [82]. However, the 5-week culture of HPs over undiluted CD38-transfected stromal cells caused an unexpected decrease in LTC-IC output (by 90%). This decrease was due to the autocrine Cx43-CD38-based interaction leading to a high Ca^2+^-induced production of interferon γ in the undiluted stromal cells, a potent hemopoiesis-inhibiting cytokine that remarkably decreased LTC-IC growth in a paracrine fashion. On the contrary, when using a mixed CD38^+/−^ stroma (diluted in a proportion of 1:10), a fivefold increase in LTC-IC output over controls was found at the end of the 5 weeks of coculture, and the IFN-γ concentration in the conditioned medium did not increase significantly [82].

The fact that BM stromal cells express ectocyclase CD157 instead of CD38 suggests the development and characterization of two CD157^+^ stromal cell lines and establishes the effect of these feeders on long-term HP cultures [41]. These CD157^+^ cells, with an ADPRC activity 50-fold lower than the undiluted CD38^+^ cells, resulted in a significantly enhanced colony output, starting from the second week of coculture. Experiments with additions to the medium of NAD^+^-glycohydrolase, ADPRC from Aplysia californica (converting NAD^+^ to cADPR), cADPR antagonists, or NTs inhibitors, demonstrated the concentrative nature of the influx of cADPR generated by feeder cells into HPs. Specifically, the NBMPR/dipyridamole-inhibitable concentrative cADPR/NT accomplishes the powerful task of providing transport into HPs of cADPR at the sub-nanomolar concentrations found in the medium of co-cultures.

The mechanisms and the roles of ADPRC (notably CD157 in the model of BM microenvironment), Cx43, and a high-performance concentrative cADPR transporter were also confirmed in vivo using transplanted NOD/SCID mice [83]. Two different protocols were followed: A) an in vitro priming of cord-blood-derived mononuclear cells (CB-MNC) with a micromolar cADPR for 24 h, followed by two sequential steps of transplantation into irradiated mice (primary and secondary transplants), lasting 20 days and a further 60 days, respectively; B) the co-infusion of 15 × 10^6^ CB-MNC together with as few as 10^6^ sublethally irradiated CD38^−^ (controls) or CD38^+^ murine stromal cells. With both protocols the terminal expansion of CD34^+^ HPs was significantly increased over the controls, not receiving cADPR-primed cells (A) or receiving CD38^−^ stromal cells (B). Thus, the expansion factor of CD34^+^ HPs was 16.5 vs. 2.3 for mice transplanted with cADPR-primed cells (A), and 57.0 vs. 16.7 for the co-infusion experiments. These results demonstrate an enhanced proliferation of committed human progenitors and the expansion of human multipotent stem cells (HSC), with increased long-term engraftment [83].

The high efficiency of the B protocol underlines the important role of the protracted and localized exposure of the HPs to the nanomolar concentrations of cADPR in the BM microenvironment. This finding reveals the in vivo performance of the CD157 ADPRC/NAD^+^/cADPR/[Ca^2+^]_i_ paracrine system, which leads to expansion of long-term repopulating cells, and confirms in vivo the highly concentrative power of the NBMPR- and dipyridamole (DP)-inhibitable CNT demonstrated by the results of in vitro studies [41].

### 4.5. CD157^+^ Paneth Cells/Intestinal Stem Cells

**Figure 7:**
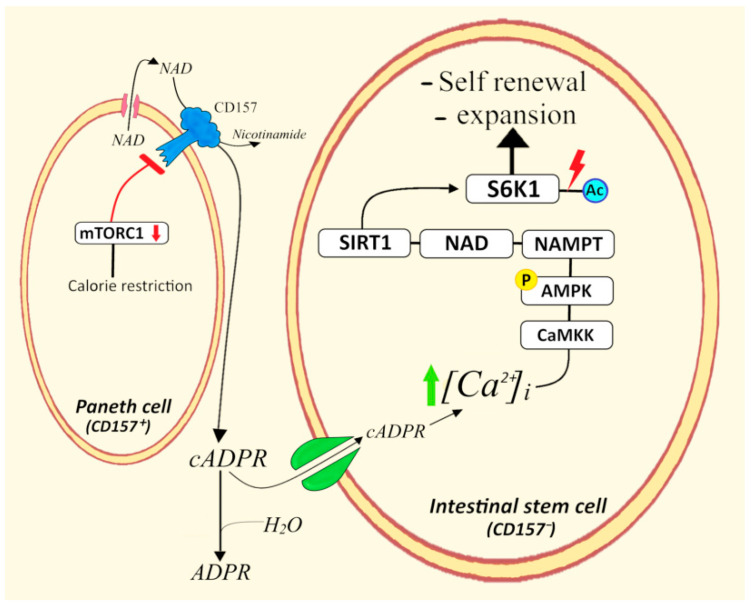
Role of Paneth cells in the Cyclic ADP-ribose-mediated expansion of intestinal stem cells during calorie restriction. mTORC1, mammalian/mechanistic target of rapamycin complex 1; NAMPT, Nicotinamide phosphoribosyltransferase; S6K1, Ribosomal protein S6 kinase 1; CaMKK, Calcium/calmodulin-dependent protein kinase kinase; AMPK, AMP-activated protein kinase; Ac, acetylation; P, phosphorylation.

The expanding function of cADPR on stem cells via paracrine mechanisms is not unique to HPs. Recently, L. Guarente investigated the mechanisms of calorie restriction (CR) in the mouse intestine and found that the intestinal niche responds to CR through an intercellular crosstalk between Paneth niche cells and intestinal stem cells (ISCs) [84,85]. Under conditions of CR, Paneth cells showed decreased signaling of the mammalian target of rapamycin complex 1 (mTORC1), which translated into induction of the ectocyclase CD157 and increased generation and release of cADPR. The ISCs in the niche responded to Paneth-cells-generated cADPR with a cascade of [Ca^2+^]_i_-CaMKK-initiated events that include in sequence: AMP kinase (AMPK) stimulation, overexpression of the rate-limiting enzyme of NAD^+^ biosynthesis (nicotinamide phosphoribosyl transferase, NAMPT), activation of the NAD^+^-consuming enzyme SIRT1, SIRT1-catalyzed deacetylation of the C terminus of S6K1 (Ribosomal protein S6 Kinase), activation of S6K1 through mTORC1 phosphorylation [84]. The stimulation of this regulatory pathway enhanced ISCs renewal instead of their differentiation (Figure 7). The steps of the pathway were identified by several experimental approaches. Notably, Yilmaz et al. [86] reported an increase in the number of ISCs induced by the decrease in mTORC1 signaling in Paneth cells, while the mTORC1 inhibitor rapamycin blocked the expansion of ISCs promoted by CR. The opposite effects of CR on mTORC1 activity in Paneth cells (downregulation) and in ISCs (upregulation) indicated that the paracrine activity of cADPR explains how ISC, unlike Paneth cells, are insulated from directly sensing CR.

More recently, Guarente’s team was able to demonstrate that CR and aging induce opposite effects on the two major cell types of the intestinal niche. Thus, during aging there was a decline in ISCs number and functional activity, while Paneth cells showed no decline in number nor in their fraction to foster ISCs. The essential role of NAD^+^ in the signaling pathway activated in the ISCs by cADPR produced and released by Paneth cells prompted Igarashi et al. [87] to investigate whether the supplementation of the NAD^+^ precursor NR (Nicotinamide Riboside) has positive effects on both the number of ISCs and the rescue of functional defects. Indeed, the aging of mice resulted in a decrease in ISCs self-renewal and increased ISC differentiation. Moreover, crypts from old mice formed fewer organoid colonies and showed a decreased number of buds, compared to those from young mice. A functional ex vivo assay demonstrated that the Paneth cells from old mice were as efficient as young Paneth cells in stimulating colony formation, while a functional defect was instead observed in old ISC cells. The supplementation of crypts from young and old mice with NR rescued the decrease in colony formation in aged mice. Additionally, NR rescued repair defects in the aging gut. The NR-induced NAD^+^ replenishment was blocked by the mTORC1 inhibitor rapamycin or by a SIRT1 inhibitor, confirming the cADPR-dependent NAD^+^/SIRT1/mTORC1 pathway in ISCs as a mechanism for rejuvenating the aged gut.

## 5. Cytotoxic T Lymphocytes and CD38-Mediated Adenosine Generation in Tumor Cells

Research on CD38 flourished a few years ago when it became clear that its ectocellular activity can initiate a multi-step pathway leading to adenosine generation in the extracellular environment and especially in the TME. Thus, in addition to a canonical adenosine-generating pathway starting from extracellular ATP and involving the ecto-nucleoside triphosphate phosphohydrolase converting ATP to ADP and to AMP (CD39), followed by the 5′-nucleotidase CD73 converting AMP to adenosine [88], a novel noncanonical pathway was proposed and shown to start from extracellular NAD^+^ as a precursor [44]. This alternative route is opened by the conversion of NAD^+^ to cADPR and ADPR, catalyzed by CD38 (arguably type II CD38, but likely also CD157), which progresses to AMP by conversion of ADPR, is catalyzed by the ecto-nucleotide pyrophosphatase/phosphodiesterase CD203a (or PC-1), and terminates with adenosine generation by CD73 [44,45,46,47,48,49,50]. Both adenosinergic pathways may operate through either a continuous mechanism (with all ectoenzymes expressed by a single cell type) or in a discontinuous fashion (with the involvement of related ectoenzymes on different cells, as occurs in specific cellular microenvironments, e.g., TME [44]).

This situation recapitulates the two major physiological functions of extracellular cADPR, i.e., calcium mobilizer and a metabolic precursor of adenosine. Accordingly, the impact of CD38 in immuno-oncology has substantially advanced in recent years, although the precise identification of its mechanisms is still elusive and incomplete. The reason for the present limitations is the pleiotropy of biochemical events downstream of the CD38/NAD^+^/cADPR/[Ca^2+^]_i_ system and of the CD38/NAD^+^/Adenosine pathway that is often intertwined. For instance, the overexpression of CD38 in several solid and hematological tumors can lead to growth and metastatic dissemination through any of the following redundant effects: (i) decline in NAD^+^ levels, both intracellular and extracellular [89,90,91,92]; (ii) hypertrophy of the alternative adenosinergic pathway in the TME and especially in tumor cells, with consequent weakening or suppression of CD8^+^ T cell function; (iii) [Ca^2+^]_i_-related mechanisms of cADPR in tumor cells, including the downregulation of the tumor suppressor KEAP1 and parallel upregulation of the NRF2 oncogene [93]. A further uncertainty, not yet clarified, concerns the likely reinforcement of the CD38-mediated mechanisms by the other ecto-ADPRC CD157, e.g., in multiple myeloma (MM) and in pleural mesothelioma cells [47,94].

Collectively, the wealth of findings on these ectoenzymes has led from a purely descriptive definition of markers of individual cell types to the elucidation of specific molecular mechanisms in physiology and pathology. Consistently, high levels of CD38 have been found in many tumors, indicating therapeutic approaches, such as the use of CD38^−/−^ model mice and CD38 inhibitors to inhibit the progression of glioma [95], lung adenocarcinoma [93] and the outgrowth of primary melanoma [96]. The drug molecules first proposed for MM include NAD^+^ analogs, 4-amino-quinolines (78c) and flavonoids (quercetin, apigenin, luteolinidin, kuromanin, rhein) [52,92].

The importance of CD38 ectoenzymatic activities in oncology has recently emerged from the finding that CD38 overexpression is a major mechanism of the acquired resistance of a number of tumors treated with immune checkpoint inhibitors (ICI), specifically PD-1/PD-L1 blockade. These blocking antibodies have revolutionized antitumor therapies by disrupting the PD-1/PD-L1 interaction [97]. Unfortunately, however, not all ICI-treated patients respond to ICI and a more accurate selection of patients is urgently needed [98]. Specifically, novel approaches to elucidating mechanisms of innate and acquired resistance are critically required [53]. A breakthrough in the field was obtained by Don L. Gibbons and his team, who reported that mouse models of tumors (melanoma and lung cancer), when treated for a long period with anti-PD-1 or anti-PD-L1 therapy, developed acquired resistance that is causally related to CD38 upregulation [51]. This was mediated by all-trans-retinoic acid (ATRA) and Interferon β (IFNβ) in the TME. Further experiments showed that CD38 overexpression in tumor cells resulted in the suppression of CD8^+^ T cell cytotoxic function through adenosine receptor signaling both in vitro and in vivo. Analyses of large data sets of human tumors also showed that CD38 is at a moderate to high expression in a significant fraction of patients with an active immune cell infiltrate in the TME. Therefore, CD38 seems to be upregulated through the inflammatory activity in the TME [51,52,53].

Based on these findings, therapy combination experiments were performed on mice tumor models. Specifically, combining an anti-PD-L1 antibody and an anti-CD38 antibody suppressed primary tumor growth and metastases more than either treatment alone [51]. Combination therapy with anti-PD-L1 antibody and the flavonoid rhein (an uncompetitive CD38 inhibitor [92]) dramatically inhibited tumor growth. Using PD-L1 KO cells and the treatment of rhein alone completely abrogated the tumors. The effect of rhein seems to open the way to further investigations with other inhibitors of CD38 enzymatic activities in view of possible pharmacological applications of CD38 overexpression in cases of ICI therapies [51]. Finally, the combination of anti-PD-L1 antibodies and Adenosine 2 receptor antagonists significantly improved antitumor effects, confirming the remarkable role of adenosine signaling in patients developing CD38 overexpression as a mechanism of acquired resistance to ICI therapies [51].

**Figure 8:**
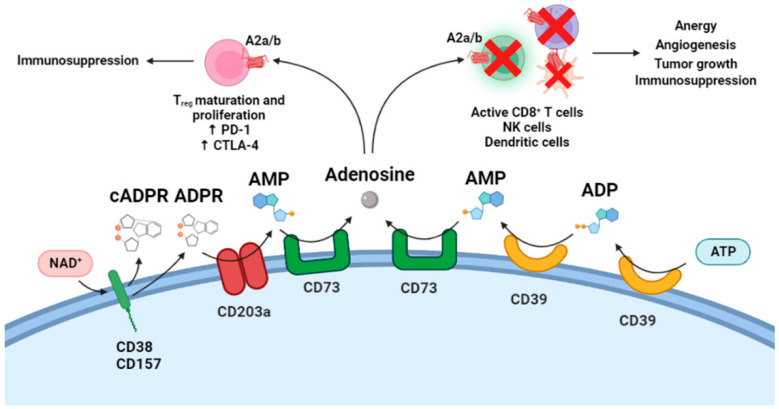
Schematic representation of adenosine production from NAD+ and ATP and its different effects on immune cells. A2a/b, Adenosine A2a/A2b receptors; CTLA-4, Cytotoxic T-Lymphocyte Associated Protein 4; PD-1, Programmed cell death protein 1.

In conclusion, this investigation elucidated a new CD38-mediated mechanism of immune evasion in ICI-treated patients based on the paracrine exchange in the TME of extracellular adenosine between tumor cells and immune cells [51]. These results hold promise for the development of new therapies that aim to treat ICI-resistant tumors by increasing the number or responder patients at the expense of non-responding patients [53]. Figure 8 illustrates extracellular adenosine production from NAD^+^ and ATP and summarizes adenosine effects on immune cells.

## 6. Conclusions and Perspectives

In several tissue microenvironments (trachea, fibroblasts, astrocytes/neurons, intestine, bone marrow), a paracrine interaction exists between NAD^+^-releasing, cADPR-generating stroma cells and cADPR-responsive parenchymal-like cells. This intercellular interaction envisages the following roles [34]:

- NAD^+^_e_ as a hormone.
- cADPR_e/i_ as a second messenger.
- [Ca^2+^]_i_ as a third messenger.

A hormone-like behavior of extracellular NAD^+^ (around 50–100 nM in plasma and extracellular fluids [32]) is strongly supported by its regulated release from a number of cell types through Cx43 hemichannels and is strengthened by the finding that NAD^+^ is a ligand and an agonist of the P2Y11 metabotropic purinoceptor in human granulocytes [99]. This result compares with the observation that NAADP^+^ is also an agonist of the P2Y11 purinergic receptor in astrocytoma cells [100].

**Figure 9:**
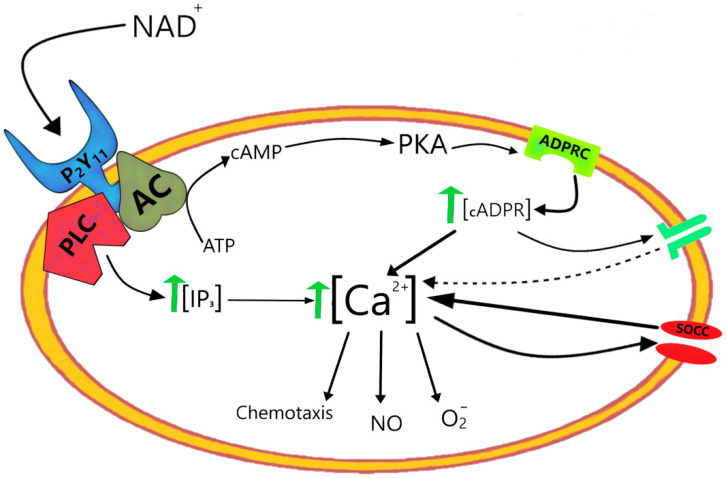
Dual roles of NAD^+^ as an agonist of P2Y11 purinergic receptor and a cADPR precursor for the [Ca^2+^]_i_-mediated mechanisms of granulocyte activation. PLC, Phospholipase C; AC, Adenylyl cyclase; ADPRC, ADP-ribosyl cyclases; SOCC, Store-operated calcium channels.

P2Y11 is the only purinergic receptor coupled to both phospholipase C (PLC) (generating IP3) and adenylate cyclase (generating cAMP) (Figure 9). The latter transduction pathway stimulates via cAMP/PKA the ADPRC activity of granulocyte CD38 with increased formation of cADPR. The IP3- and cADPR-mediated increases in [Ca^2+^]_i_ responses were transient and sustained, respectively, and the latter increase was causally related to the NAD^+^-triggered activation of granulocytes, i.e., superoxide and nitrite production and enhanced chemotaxis [101]. The scheme of Figure 9 demonstrates the uncommon dual role of NAD^+^ in increasing the granulocyte responses, as an ectocellular agonist of P2Y11 and as an intracellular metabolic precursor in the signal-transducing pathway downstream of NAD^+^-P2Y11 binding [99].

Another report of NAD^+^ as a hormone-like signaling molecule targeting murine lymphocytes concerns the competing roles in NAD^+^ consumption between CD38 and ADP-ribosyltransferase-2 (ART2) which catalyzes ADP-ribosylation of the P2X7 ionotropic purinergic receptor and of other important cell surface proteins [102]. Indeed, ADP-ribosylation of P2X7 at low extracellular concentrations of NAD^+^ resulted in the rapid death of T cells [103].

It is remarkable that the paracrine effects of the NAD^+^/cADPR system are mediated by the same NAD^+^ and cADPR transporters responsible for the traffic of signal metabolites involved as a cell-autonomous mechanisms, i.e., in an autocrine fashion [32,34]. This seems to facilitate search for identifying and characterizing other intercellular systems involved in pathophysiological conditions. The clearest example of this approach is the profitable use of transwell co-cultures, where analyses of metabolites in the medium and comparative evaluation of biochemical differences between cADPR-generating and cADPR-responsive cell types are very informative.

Nevertheless, several questions remain unanswered and require new strategies focusing on unicellular versus multicellular systems. For instance, there is only one report on the possible roles and mechanisms of NAADP^+^ as a signal metabolite connecting different cell systems, specifically pancreatic beta cells and adipocytes [104]. Moreover, whether cADPR acts through the same mechanisms within distinct cell types is still not completely defined. For example, the field of intracellular cADPR-binding proteins is far less known than the identity and properties of NAADP^+^-binding proteins [105,106,107,108] and is so far limited to Glyceraldehyde 3-P dehydrogenase (although through only partially known mechanisms) [109] and to the immunosuppressant FK506-binding protein 12.6 [110].

The simultaneous occurrence of autocrine and paracrine cADPR-mediated events (see the astrocytes/neurons and the stroma/HPs cell systems) requires a deeper collection of data on NAD^+^ metabolism (synthesis versus consumption) and on the cADPR/Ca^2+^ system at the same time. Likewise, attention should be paid to better clarifying the identity and the functional efficiency of cADPR/nucleoside transporters as essential players of cADPR utilization in the same cell and in non-cADPR-generating cells [32].

The paracrine cell systems described in this review underline the specific functions of the two major ecto-ADPRCs, type II CD38 and CD157, which produce and supply cADPR to neighboring cells. Related to this point, more advanced information on the comparative properties of the two ectoenzymes is needed, beyond the substantially higher cADPR-producing activity of CD38, especially possible differences in their regulation in different cell types and tissues [77,78], including yet undetermined mechanisms of gene expression. Limited but promising examples concern the distinct roles and mechanisms of CD38 and CD157 in (i) inducing an immunosuppressive TME in solid tumors [96], (ii) showing different substrate properties in the brain that are apparently associated with specific psychiatric symptoms and psychological disorders [111].

Arguably, other non-ectocellular ADPRCs, such as type III CD38 and SARM1, should not be directly involved in the production and export of cADPR to adjacent cells. In any case, the enzymatic mechanisms of cADPR generation, although not directly relevant to cADPR “secretion”, might play some role, e.g., by distinctively fueling the activity of cADPR/nucleoside transporters in certain conditions. This possibility emerges from a very recent study suggesting that decreased NAD^+^ levels are correlated with dysfunction of T cells, but the deficiency of CD38 is not sufficient to rescue NAD^+^ in tumor-infiltrated T cells, and the mechanisms of compensation include an increased expression of the non-ectoenzymatic ADPRCs CD157 and SARM1 [112].

Therefore, there is no doubt that type III CD38 and SARM 1 require accurate investigations concerning their regulatory properties (e.g., by CIB1 protein for the former and NMN for the latter), despite the same subcellular localization and especially the surprising identity of substrates (NAD^+^, NADP^+^) and products (cADPR, ADPR, NAADP^+^).

Finally, the recent groundbreaking results on the key roles of type II CD38 as a driver of acquired resistance in some immune checkpoint inhibitors (ICI)-treated patients through the paracrine production of immunosuppressant adenosine have opened a new, far-reaching field. In fact, there are only limited and vague hints as to which mechanisms are able to distinguish ICI responders from non-responders, and there is currently an unmet and urgent need to reach this goal, i.e., a rational selection of candidate patients or, even further, the acquisition of appropriate personalized therapies for several tumors [51,52]. A better understanding of the possible mechanistic connections between cADPR and adenosine would certainly help predict the success or failure of ICI treatments in oncological patients. This might greatly benefit from an advanced knowledge of cell types present in the TME and would specifically require some new approaches to the possible targeting of cADPR-mediated effects versus adenosine/adenosine receptors in selected and reciprocally interacting cell types, especially immune cells and neoplastic cells [98].

## Data Availability

Not applicable.
